# Time Window for Acute Stroke Treatment: Current Practice in King Abdullah Medical City Specialist Hospital in Makkah, Saudi Arabia

**DOI:** 10.7759/cureus.28878

**Published:** 2022-09-07

**Authors:** Amal Alkhotani, Yousef Alharbi, Hadeel Alghamdi, Hadeel Alshareef, Jannat A Abdulmuttalib, Amal Alsulami, Abdulaziz Alharbi

**Affiliations:** 1 Neurology, King Abdullah Medical City, Mecca, SAU; 2 Medicine, Umm Al-Qura University, Faculty of Medicine, Mecca, SAU; 3 Medicine, Unaizah College of Medicine and Medical Sciences, Qassim University, Unaizah, SAU; 4 Medicine and Surgery, Umm Al-Qura University, Faculty of Medicine, Mecca, SAU; 5 Pharmacy, Qassim University, Unaizah, SAU

**Keywords:** tissue plasminogen activator, emergency, interval time, delay treatment, tpa treatment, stroke

## Abstract

Introduction

Stroke has become one of the most severe causes of long-term neurological impairment and disability and is considered one of the leading causes of mortality worldwide. This study aimed to determine time delays in stroke patients from symptoms onset to treatment with tissue plasminogen activator (tPA) initiation in King Abdullah Medical City Specialist Hospital, Makkah, Saudi Arabia.

Patients and methods

We reviewed 81 patients who suffered from acute stroke. The data were collected from patients’ electronic and paper files. Patients were divided into two main categories based on interval time from recognition of symptoms to tPA treatment. Patients were divided into early treatment, if the duration was less than or equal to 120 minutes, and delayed treatment, if the interval time was more than 120 minutes.

Results

Nearly two-thirds (64.2%) were males, and more than half (5.6%) were in the older age group (>65 years). Patients who underwent thrombectomy were 7.4%. The mean value of the National Institutes of Health Stroke Scale (NIHSS) score was 10.7 (SD: 7.14). The mean time from symptoms onset to arrival at the hospital was 82.4 (SD: 44.1) minutes, while the total time from recognition of symptoms to tPA treatment was 154 (SD: 50.8) minutes. The prevalence of patients with delayed treatment was 72.8%, and the rest were assumed to have early treatment (27.2%). None of the socio-demographic variables were predicted to influence delayed treatment.

Conclusion

A significant number of patients were delayed in treatment. Patients' socio-demographic data and NIHSS scores seem to have no significant effect on delayed treatment. Further research is needed to establish the delay in time for pre-hospital and in-hospital treatment of stroke patients.

## Introduction

Stroke has become one of the most severe causes of long-term neurological impairment and disability and is considered one of the leading causes of mortality worldwide. It is defined as the death of brain tissue and deterioration of its function by loss of blood supply to the brain, triggered by ischemia (lack of blood flow), blockage (either thrombosis or embolism), or a hemorrhage. Depending on the disease severity and type of stroke, it can leave the patient with dysfunction in different aspects, including physical, psychological, social, and cognitive functions. According to the World Health Organization (WHO), about 15 million people all over the world suffer from strokes each year. Among these, five million die, and another five million are permanently disabled [1].

Over the past decade, there was only one study that says that the crude incidence rate for the first-ever incidence of stroke in Saudi Arabia was 29.8/100,000/year. They also reported that ischemic strokes stand for about 69% as the most common type and subarachnoid hemorrhage (SAH) was extremely rare (1.4%). Another study conducted between 1982 and 1992, by Al Rajeh and Awada, in a hospital that exclusively treated the Saudi Arabian National Guard community rated the crude annual incidence rate at 43.8 per 100,000 [2].

Studies reported that old age, high blood pressure, prior stroke or transient ischemic attack (TIA), diabetes, high cholesterol, tobacco smoking, and atrial fibrillation were the significant risk factors for stroke [1].

There are a variety of signs and symptoms of stroke, and abrupt onset of difficulty in speech and focal weakness are the most critical physical symptoms of a stroke. Acute onset of unilateral weakness is also a common finding. Recognition of Stroke in the Emergency Room (ROSIER) is one of the validated instruments; in the United Kingdom, it correctly classified 90% of patients with stroke. There are two conditions that might mimic the signs and symptoms of stroke: hypoglycemia and seizures. Imaging is essential for the diagnosis of stroke, and magnetic resonance imaging (MRI) has higher sensitivity than CT; therefore, it is a better detector of strokes [3].

Thrombolytic therapy has been approved for the treatment of acute stroke based on a meta-analysis study, which was conducted on 2775 patients from 18 different countries to see if early administration of nine intravenous recombinant tissue plasminogen activators (rt-PA) is associated with a better outcome. The results of this study showed the earlier the rt-PA is administered to patients with signs and symptoms of stroke, the better the outcome, especially if the treatment is given within 90 minutes. Stroke patients who have been given rt-PA beyond three hours still have some potential benefits. However, this potential has risks [4].

Thrombolytic therapy is dependent on the time of the intervention. Thus, early recognition and hospitalization of patients with stroke are very crucial for the overall outcome and benefit of the treatment. In 1997, a study was conducted in New Jersey, USA, on a total of 553 patients about the factors that might delay the signs of stroke to the arrival at an emergency department (ED), and the time from arrival at the ED to the patient's evaluation. Results state that 32% of patients with acute stroke signs or symptoms arrived at the ED within 1.5 hours, 46% of the patients arrived within three hours, and 61% within six hours. Delay in time was associated with sex, race, and transportation mode [5].

The previous study was not the only study that had been done. Multiple studies were conducted to measure the delay in pre-hospital and hospital settings. One of them shows that the median time from symptom onset to ER arrival was 2.6 hours (interquartile range: 1.2-6.3). The median time from ER arrival to CT scan completion was 1.1 hours (0.7-1.8), and the total delay time (from symptom onset to CT scan completion) had a median of 4.0 (2.3-8.3) hours. Patients who arrived by emergency medical services (EMS) had significantly shorter pre-hospital delay times and times to CT scans. In the same study, age, race, sex, and educational level did not appear to affect pre-hospital delay times [6]. Another study done on different populations shows that the median pre-hospital delay was 151 minutes (range: 5-9590 minutes). The median time interval between ED admission and neurological consultation was 27 minutes (range: 0-2165 minutes). The median time between ED admission and CT/MRI imaging was 108 minutes (range: 1-6868 minutes) [6].

Another study was done in Switzerland between January 1, 2000, and April 30, 2002, on 597 patients diagnosed with acute stroke to determine the time before giving intra-arterial thrombolysis aiming to improve the management of these patients. This study was done on three groups of patients: the first group came to the ER directly, the second was referred from hospitals that do not have a CT scan, and the third was from a hospital that had a CT scan. The mean time to arrival in the ED was 99 minutes for patients who were admitted directly with a mean of 234 minutes delay from symptom onset to management. For patients who were referred from centers that do not have a CT scan, the mean time to ED arrival was 127 minutes with a mean of 269 minutes delay from symptom onset to treatment. In the last group of patients, the mean time of ED arrival delay was 210 minutes with a mean of 302 minutes from symptoms onset to management [7].

In 2002, a multi-centric prospective study was conducted in the United Kingdom on 739 patients to investigate the delay of presentation to the hospital and the early evaluation of patients with stroke. The results of this study state that 37% of patients arrived within three hours, whereas 50% of them arrived within six hours. Delays in patients who used the emergency service were two hours and three minutes. On the other hand, the patients who were referred by general practitioners took seven hours and 12 minutes. In conclusion, we can reduce the delays of patients with suspected stroke by increasing the use of the ED [8].

Between June 2004 and October 2005, a prospective observational study was conducted in Taiwan on 129 patients that had an ischemic stroke to know the time and elements that led to pre-hospital and ED delays in the management of these patients. This study showed that the median time from the onset of symptoms to reaching the ED was 71 (mean ± SD: 82.7 ± 57.7) minutes. Moreover, the median times from ED arrival to neurologic consultation was 10 (11.3 ± 9.9) minutes, from ED arrival to CT scan was 17 (9.6 ± 11.3) minutes, from ED arrival to ECG was 14 (23.3 ± 55) minutes, and from ED arrival to laboratory data completion was 39 (44.4 ± 24.5) minutes [9].

Pre-hospital delays take up a more extensive part of total delay time. Nevertheless, there has been a decrease in delay time over the past years. Based on studies of many different population groups (65 groups), there was a 10.2% annual decrease in hours per year from ED arrival to a neurological evaluation (p = 0.23 based on 16 population groups) and a 10.7% annual drop in hours per year for delay time from ED arrival to initiation of CT (p = 0.11 based on 23 population groups) [10].

According to a 2020 study conducted in Saudi Arabia, the majority of emergency and medical staff doctors had never administered tissue plasminogen activator (tPA) and were never involved in its administration to a stroke patient [11]. In another study, the following success factors were crucial to the timely administration of tPA in stroke therapy: (1) extensive knowledge, skill, and a positive attitude on the part of nurses toward stroke care; (2) organized systems and technology for managing stroke patients in the emergency room; (3) teamwork; and (4) clearly defined nursing roles for assessing and monitoring stroke patients. The hurdles, on the other hand, include everything related to the patient, staffing, doctor, education, resource, and teamwork [12]. In Makkah, Saudi Arabia, a recent cross-sectional study was conducted in 2022 to evaluate the late hospital arrival of patients with acute ischemic stroke. It was discovered that 45% of patients presented late and 55% of patients presented early [13].

Rationale

Stroke is correlated with a high mortality rate and long-term neurological impairment and disability. “Time” is considered the most crucial factor that determines the prognosis. However, there are few research studies in Saudi Arabia that studied the importance of timing in acute stroke management. This research aimed to know the current situation in King Abdullah Medical City Specialist Hospital, Makkah, Saudi Arabia, which is a center of stroke in the Makkah healthcare system in regard to the acute stroke time window. The findings of this study will help healthcare practitioners to know their time window and compare it to the international guidelines. Also, this study will increase awareness for the general population of stroke signs and symptoms, and doctors of the importance of giving the tPA within the window.

## Materials and methods

This retrospective cohort study was conducted at King Abdullah Medical City Specialist Hospital in Makkah, Saudi Arabia. The study sample included all patients diagnosed with acute stroke and who received tPA from the 1st of July 2019 to the 31st of December 2021. The exclusion criteria were any patients with neurological deficits that were not caused by acute stroke and patients with hemorrhagic stroke. Patients who died upon arrival or during transportation to the hospital, and those who presented late (>4.5 hours of first symptoms onset) were also excluded.

Data collection was done using medical records and the administrative data of acute stroke patients were collected. A pre-designed checklist was prepared to collect data about patients' demographics and social data, clinical presentation, time of onset of the first symptom, time of seeking medical help, time of ED arrival, time of ED clinical examination, time of consultation, time of CT interpretation, and time of therapeutic interventions.

Patients were classified as having delayed treatment (>120 minutes) and having an early treatment (≤120 minutes) based on the same cutoff point used in previous studies [6,14].

Statistical analysis

Data were statistically analyzed using the SPSS program version 26 (IBM Corp., Armonk, NY). To investigate the association between the variables, Fischer's exact test was applied to qualitative data that were expressed as numbers and percentages. Quantitative variables were expressed as mean and standard deviation (mean ± SD) and the association between non-parametric data was assessed by the Mann-Whitney U-test. Statistical significance was defined as a p-value of less than 0.05.

## Results

This study involved 81 patients who suffered from a stroke. As seen in Table 1, 50.6% were aged more than 65 years old with a higher incidence of stroke in males (64.2%). Only 2.5% were living alone, while 77.8% were able to recognize the symptoms by themselves. First-time incidence of stroke happened to more than three-quarters of the patients (76.5%). Documentation of onset was known to all patients while only 8.6% were able to recognize symptoms after sleep. The majority of patients were diagnosed with anterior stroke (75%), while 25% were diagnosed with posterior stroke. The prevalence of patients who underwent thrombectomy was 7.4%. In addition, the mean National Institutes of Health Stroke Scale (NIHSS) score was 10.7 (SD: 7.14).

**Table 1:** Baseline characteristics of the patients NIHSS: National Institutes of Health Stroke Scale.

Study variables	N (%)
Age group	
≤65 years	40 (49.4%)
>65 years	41 (50.6%)
Gender	
Male	52 (64.2%)
Female	29 (35.8%)
Home steady living	
Alone	02 (02.5%)
Not alone	62 (76.5%)
Unknown	17 (21.0%)
Recognition of symptoms	
By self	63 (77.8%)
Not by self	18 (22.2%)
Frequency of sickness	
First	62 (76.5%)
Recurring	19 (23.5%)
Documentation of onset time	
Known	81 (100%)
Unknown	0
Symptoms recognized after sleep	
Yes	07 (08.6%)
No	74 (91.4%)
Patient had thrombectomy	
Yes	06 (07.4%)
No	75 (92.6%)
NIHSS score (mean ± SD)	10.7 ± 7.14

In Table 2, the mean time (minutes) from symptoms onset to arrival at the hospital, examination by the emergency doctor, the first sample taken, CT scan done, examination by specialists, and tPA treatment in minutes were 82.4, 92.9, 97.3, 128.2, 134.9, and 156.8, respectively. The total time from recognition to tPA treatment was 154.4 (SD: 50.8) minutes.

**Table 2:** Descriptive statistics of the interval from the recognition of symptoms to tPA treatment tPA: tissue plasminogen activator.

Interval time (minutes)	Mean ± SD	Median	Min	Max
Arrival at the hospital	82.4 ± 44.1	90.00	5.00	165.0
Examination by the emergency doctor	92.9 ± 45.4	97.00	7.00	180.0
First sample was taken	97.3 ± 46.6	102.0	7.00	215.0
CT was done	128.2 ± 49.9	130.0	30.0	231.0
Examination by a specialist	134.9 ± 49.1	140.0	35.0	240.0
tPA treatment was done	156.8 ± 47.8	165.0	60.0	255.0
Total time from recognition to tPA treatment	154.4 ± 50.8	165.0	10.0	255.0

**Figure 1:**
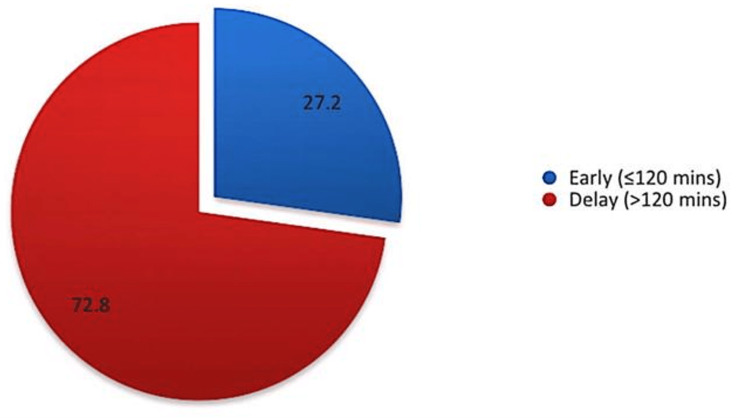
Total interval time from recognition of symptoms to tPA treatment tPA: tissue plasminogen activator.

Figure 1 shows the stratified total interval time from recognition of symptoms to tPA treatment. It can be observed that 72.8% were classified as delayed treatment while the rest (27.2%) were classified as early intervention.

When measuring the relationship between delayed tPA treatment and the baseline characteristics of the patients (Table 3), it was found that there were no significant differences between delayed tPA treatment according to age group (p = 0.804), gender (p = 0.608), recognition of symptoms (p = 0.371), frequency of sickness (p = 0.569), symptoms recognized after sleep (p = 0.667), those who underwent thrombectomy (p = 0.661), and NIHSS score (p = 0.527).

**Table 3:** Relationship between delayed tPA treatment and the baseline characteristics of the patients ^§^ P-value has been calculated using Fischer's exact test. ^‡^ P-value has been calculated using the Mann-Whitney U test. tPA: tissue plasminogen activator; NIHSS: National Institutes of Health Stroke Scale.

Factor	Early, N (%) (n = 22)	Delayed, N (%) (n = 59)	P-value^§^
Age group			
≤65 years	10 (45.5%)	30 (50.8%)	0.804
>65 years	12 (54.5%)	29 (49.2%)	
Gender			
Male	13 (59.1%)	39 (66.1%)	0.608
Female	09 (40.9%)	20 (33.9%)	
Recognition of symptoms			
By self	19 (86.4%)	44 (74.6%)	0.371
Not by self	03 (13.6%)	15 (25.4%)	
Frequency of sickness			
First	18 (81.8%)	44 (74.6%)	0.569
Recurring	04 (18.2%)	15 (25.4%)	
Symptoms recognized after sleep			
Yes	01 (04.5%)	06 (10.2%)	0.667
No	21 (95.5%)	53 (89.8%)	
Patient had thrombectomy			
Yes	02 (09.1%)	04 (06.8%)	0.661
No	20 (90.9%)	55 (93.2%)	
NIHSS score (mean ± SD)	11.3 ± 7.03	10.4 ± 7.23	0.527^‡^

## Discussion

Time is crucial for the successful treatment and management of acute stroke. This has been discussed well in previous studies [14-19]. The present study sought to determine the time delays in stroke patients from symptoms onset to treatment with tPA initiation in King Abdullah Medical City Specialist Hospital, Makkah, Saudi Arabia. The findings of this study revealed that the mean duration of time from symptoms onset to tPA treatment was 156.4 (SD: 50.8) minutes with 72.8% classified as delayed treatment (>120 minutes) while 27.2% classified as early treatment (≤120 minutes). There were conflicting reports regarding the cutoff times between delayed and on-time treatment. For example, a study conducted by Hacke et al. [4] reported that the median time from onset to start of treatment was 243 minutes. The study concluded that the earlier rt-PA is given to stroke patients, the better the benefit, especially if started within 90 minutes suggesting a promising benefit beyond three hours but this potential might come with certain risks. In another study published in Switzerland [7], the authors indicated that the mean delay from symptom onset to treatment was 234 minutes for Bern patients (admitted directly to the Inselspital), 269 minutes for non-Bern/-CT patients (referred from a community hospital without a CT scan), and 302 minutes for non-Bern/+CT patients (referred from a community hospital after CT scan), where patients from the last group needed longer time to receive intra-arterial thrombolysis than did patients who were admitted directly or who were transferred from a hospital without having a CT scan.

Data in our study suggest that the mean time from symptoms onset to arrival to the ED was 82.4 (SD: 44.1) minutes, to ED doctor was 92.4 (SD: 45.4) minutes, first sample analysis was 97.3 (SD: 46.6) minutes, to CT scan done was 128.2 (SD: 49.9) minutes, and to the neurologic consultation was 134.9 (SD: 47.8) minutes. These findings are almost consistent with a paper conducted in Taiwan [9], showing that the mean time from symptom onset to ED arrival was 82.7 (SD: 57.7) minutes, ED arrival to CT scan was 9.6 (SD: 11.3) minutes, ED arrival to neurologic consultation was 11.3 (SD: 9.9) minutes, and to laboratory data was 44.4 (SD: 24.5) minutes.

The study further indicated that two hours of pre-hospital delay is the cutoff point for thrombolytic therapy. In our study, we included the time from onset to CT interpretation by a radiologist and documentation in the system. That resulted in the delay in presentation to CT.

However, our results are faster than that of Lacy et al. [15], showing that 32% arrived at the hospital at 1.5 hours of stroke onset, 46% arrived within three hours, and 61% within six hours. In view of this delay, the investigator discovered that patients arriving by ambulance and those who needed admission to ICU were examined sooner by the physicians. On the other hand, in a comprehensive review carried out by Evenson et al. [10], between the years 1981 to 2007, based on studies on 65% of population groups, the weighted Poisson regression indicated a 6% annual decline in hours/year for pre-hospital delay defined from symptom onset to ED arrival, and the annual decline in hours/year for delay time from ED arrival to initiation of CT scan was represented by 10.7%.

The author concluded that it is interesting to see more effective community-based intervention worldwide for the next decade and how they can improve stroke surveillance systems that will improve pre-hospital and in-hospital delays for acute stroke care.

Al Khathaami et al. stated in a prior Saudi study conducted in Riyadh that specific therapeutic methods, such as tPA, are beneficial when given within 4.5 hours after the onset of acute ischemic stroke. However, because of their tardy admission to the hospital, the majority of patients are still untreated. According to the study's findings, the patient's delayed arrival was caused by a number of factors, including the patient being alone when the stroke first occurred, not being taken to the hospital in an ambulance, being unaware of the disease they had, living a long way from the hospital, being uninformed, and having difficulty accessing healthcare services [20]. In 2020, Al Khathaami et al. estimated that rt-PA was administered to 8.6% of all acute ischemic stroke patients who presented to Saudi Arabia's tertiary care hospitals. Since alteplase must be delivered as quickly as possible, late entrance to the emergency room is the main cause of underutilization. Only 29.8% of the patients in the aforementioned trial arrived in time for such intervention [21].

Several papers indicated a significant relationship between delayed treatment according to the socio-demographic variables of the patients. For instance, in a study by Jungehulsing et al. [6], females living by themselves were significantly related to increased delays, while Chen et al. [9] documented that based on regression models, age of fewer than 65 years, being illiterate, and symptoms awakening were the significant factors associated with delay in ED presentations. In a study by Wester et al. [22], gradual onset, mild neurological symptoms, brain infarct, patients who took care of themselves, patients who lived in a large catchment area, those who visited a primary care site, and those who did not use ambulance transportation were the factors associated with increasing delay to hospital admission. Likewise, Morris et al. [23] reported that patients who arrived by EMS had significantly shorter pre-hospital delay times and times to CT scans. However, patients’ age, race, sex, and educational level appeared to not affect pre-hospital delay times. In our study, we found no significant differences between delayed treatment and the socio-demographic variables of the patients. It may be because of the limited sample size or other potential confounders involved in this study.

Thrombolytic programs have been implemented in many hospitals in Saudi Arabia [11]. Besides, the Saudi Ministry of Health had developed acute stroke screening and code route methods in accordance with Vision 2030. This approach tries to standardize emergency room and hospital acute stroke care. These procedures cover the treatment of subarachnoid hemorrhage, intracerebral hemorrhage, and acute ischemic stroke. The protocol intends to cover transfer protocols with primary stroke hospitals (PSHs) and comprehensive stroke hospitals (CSHs), decisions on the acute use of IV r-tPA for selected acute ischemic stroke patients, and medical stabilization of the stroke patient in terms of managing their blood pressure and airway [24].

Further investigations are warranted to determine the true effect of socio-demographic characteristics in the delayed treatment of acute stroke patients. Being a single-center study was a limitation of the present work, besides the small sample size. A multi-center approach would result in a better outcome and could provide a better insight into the influential factor of delayed in-hospital treatment in our region. Future studies could be done to assess the NIHSS score after the number of weeks or months among the two groups of patients. Also, data about the functional status of patients as a main public health problem could be assessed in future studies.

## Conclusions

A significant number of patients were delayed in treatment. That happened due to pre-hospital delays of patients. Also, patients' socio-demographic data along with NIHSS scores seem to have no significant effect on delayed treatment. Despite collective efforts to uphold timely stroke evaluation and treatment, the delays cannot behold. This underlines the need for effective public health programs intended to minimize time consumption when evaluating and treating patients who suffered from acute ischemic stroke. Further research is needed to establish the delay in time for pre-hospital and in-hospital treatment of stroke patients.

## References

[1] Robert AA, Zamzami MM (2014). Stroke in Saudi Arabia: a review of the recent literature. Pan Afr Med J.

[2] Al Rajeh S, Awada A (2002). Stroke in Saudi Arabia. Cerebrovasc Dis.

[3] Yew KS, Cheng EM (2015). Diagnosis of acute stroke. Am Fam Physician.

[4] Hacke W, Donnan G, Fieschi C (2004). Association of outcome with early stroke treatment: pooled analysis of ATLANTIS, ECASS, and NINDS rt-PA stroke trials. Lancet.

[5] Lacy CR, Bueno M, Kostis JB (1997). Stroke time registry for outcomes knowledge and epidemiology S.T.R.O.K.E. J Stroke Cerebrovasc Dis.

[6] Jungehulsing GJ, Rossnagel K, Nolte CH (2006). Emergency department delays in acute stroke - analysis of time between ED arrival and imaging. Eur J Neurol.

[7] Nedeltchev K, Arnold M, Brekenfeld C, Isenegger J, Remonda L, Schroth G, Mattle HP (2003). Pre- and in-hospital delays from stroke onset to intra-arterial thrombolysis. Stroke.

[8] 8] Stroke (2022). Verita Neuro. Stroke. https://veritaneuro.com/stroke-sem/?gclid=Cj0KCQjwrs2XBhDjARIsAHVymmRKPJUPgnTZJpqtR_CqPjwBQcMvN0_A0RU2CFcGz6g1aCI4lbgMEFAaAgATEALw_wcB.

[9] Chen CH, Huang P, Yang YH, Liu CK, Lin TJ, Lin RT (2007). Pre-hospital and in-hospital delays after onset of acute ischemic stroke—a hospital-based study in southern Taiwan. Kaohsiung J Med Sci.

[10] Evenson KR, Foraker RE, Morris DL, Rosamond WD (2009). A comprehensive review of prehospital and in-hospital delay times in acute stroke care. Int J Stroke.

[11] Alqwaifly M (2020). Acute stroke care practice among emergency and medicine staff in Qassim region, Saudi Arabia. Int J Adv Med.

[12] Catangui E, Baua E, Pizarro J, Almutairi AF (2020). Timely administration of thrombolytic therapy in acute ischemic stroke: an ethnographic study. Int J Nurs Clin Pract.

[13] Alkhotani AM, Almasoudi A, Alzahrani J, Alkhotani E, Kalkatawi M, Alkhotani A (2022). Factors associated with delayed hospital presentation for patients with acute stroke in Makkah: a cross-sectional study. Medicine (Baltimore).

[14] Mayer-Reichenauer M, Dachenhausen A, Bosak P (1999). Time delays in admission to a stroke unit and emergency treatment of patients with ischemic stroke. (Article in German). Dtsch Med Wochenschr.

[15] Lacy CR, Suh DC, Bueno M, Kostis JB (2001). Delay in presentation and evaluation for acute stroke: Stroke Time Registry for Outcomes Knowledge and Epidemiology (S.T.R.O.K.E.). Stroke.

[16] Harraf F, Sharma AK, Brown MM, Lees KR, Vass RI, Kalra L (2002). A multicentre observational study of presentation and early assessment of acute stroke. BMJ.

[17] Chang KC, Tseng MC, Tan TY (2004). Prehospital delay after acute stroke in Kaohsiung, Taiwan. Stroke.

[18] Derex L, Adeleine P, Nighoghossian N, Honnorat J, Trouillas P (2002). Factors influencing early admission in a French stroke unit. Stroke.

[19] Rossnagel K, Jungehülsing GJ, Nolte CH (2004). Out-of-hospital delays in patients with acute stroke. Ann Emerg Med.

[20] Al Khathaami AM, Mohammad YO, Alibrahim FS, Jradi HA (2018). Factors associated with late arrival of acute stroke patients to emergency department in Saudi Arabia. SAGE Open Med.

[21] Al Khathaami AM, Al Bdah B, Tarawneh M (2020). Utilization of intravenous tissue plasminogen activator and reasons for nonuse in acute ischemic stroke in Saudi Arabia. J Stroke Cerebrovasc Dis.

[22] Wester P, Rådberg J, Lundgren B, Peltonen M (1999). Factors associated with delayed admission to hospital and in-hospital delays in acute stroke and TIA: a prospective, multicenter study. Stroke.

[23] Morris DL, Rosamond W, Madden K, Schultz C, Hamilton S (2000). Prehospital and emergency department delays after acute stroke: the Genentech Stroke Presentation Survey. Stroke.

[24] (2019). Saudi Ministry of Health. Saudi Stroke Pathway Standards. https://chi.gov.sa/AboutCCHI/CCHIprograms/Documents/Saudi_stroke_standards_1579260606.pdf.

